# Maternal Androgens in Dominant Meerkats (*Suricata suricatta*) Reduce Juvenile Offspring Health and Survivorship

**DOI:** 10.1002/ece3.70600

**Published:** 2024-11-21

**Authors:** Kendra N. Smyth‐Kabay, Nicholas M. Caruso, Alexandra C. Stonehill, Tim H. Clutton‐Brock, Christine M. Drea

**Affiliations:** ^1^ University Program in Ecology, Duke University Durham North Carolina USA; ^2^ Department of Evolutionary Anthropology Duke University Durham North Carolina USA; ^3^ Kalahari Research Trust, Kuruman River Reserve Van Zylsrus Northern Cape South Africa; ^4^ Department of Biological Sciences University of Alabama Tuscaloosa Alabama USA; ^5^ Department of Zoology University of Cambridge Cambridge UK; ^6^ Mammal Research Institute University of Pretoria Pretoria South Africa

**Keywords:** cooperative breeding, ecoimmunology, female masculinisation, female social dominance, immunocompetence handicap hypothesis, maternal effects, reproductive and developmental trade‐offs, sexual selection

## Abstract

In oviparous vertebrates, maternal androgens can alter offspring immune function, particularly early in development, but the potential for negative health effects of maternal androgens in mammals remains unclear. We investigated the relation between maternal androgens, particularly in late gestation, and offspring health in the meerkat (*Suricata suricatta*) by comparing offspring from (a) normative dominant and subordinate matrilines, whose dams naturally express high versus lower circulating androgen concentrations, respectively, and (b) normative dominant and antiandrogen‐treated dominant matrilines, whose dams' androgen function was intact versus blocked owing to experimental antagonism of the latter's androgen receptors (using Flutamide). Foetal offspring thus experienced three different endocrine environments (‘high’, ‘lower’ and ‘blocked’ androgens) late in prenatal development. We assessed parasitism, immune function, sex steroid concentrations and survivorship in these three offspring groups, both during juvenility and early adulthood. The juvenile offspring of subordinate control and dominant treated dams generally had lower intensities of parasite infections and greater immune function than did their peers from dominant control dams—patterns not found in adult offspring, or in relation to the offspring's concurrent hormone concentrations. Survivorship to adulthood was greatest in the progeny of treated dams. Descendants of dominant female meerkats—those in the ‘high’ prenatal androgen category—suffered increased parasitism and decreased immunocompetence as juveniles, as well as reduced survivorship relative to antiandrogen‐exposed peers, providing evidence in mammals that maternal androgens can negatively impact offspring health and survival. These intergenerational, androgen‐mediated, health effects represent early costs imposed by female intrasexual competition and its associated selection pressures.

## Graphical abstract

The progeny of normative dominant female meerkats that had been exposed to the highest concentrations of maternal androgens prenatally suffered increased parasitism and decreased immunocompetence as juveniles (but not as adults), as well as reduced survivorship relative to the offspring of normative subordinate female meerkats and/or to antiandrogen‐exposed peers. These data provide the first evidence in wild mammals that naturally raised maternal androgens can negatively impact early offspring health and survival. These intergenerational, androgen‐mediated, health effects represent early costs imposed by female intrasexual competition and its associated selection pressures.

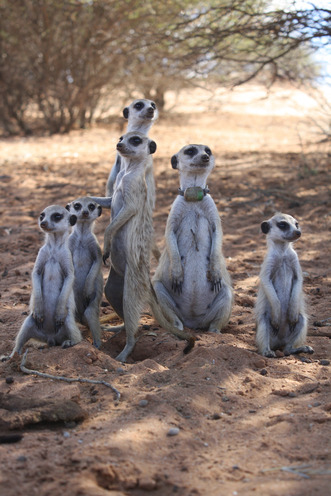

## 1 Introduction

**FIGURE 1:**
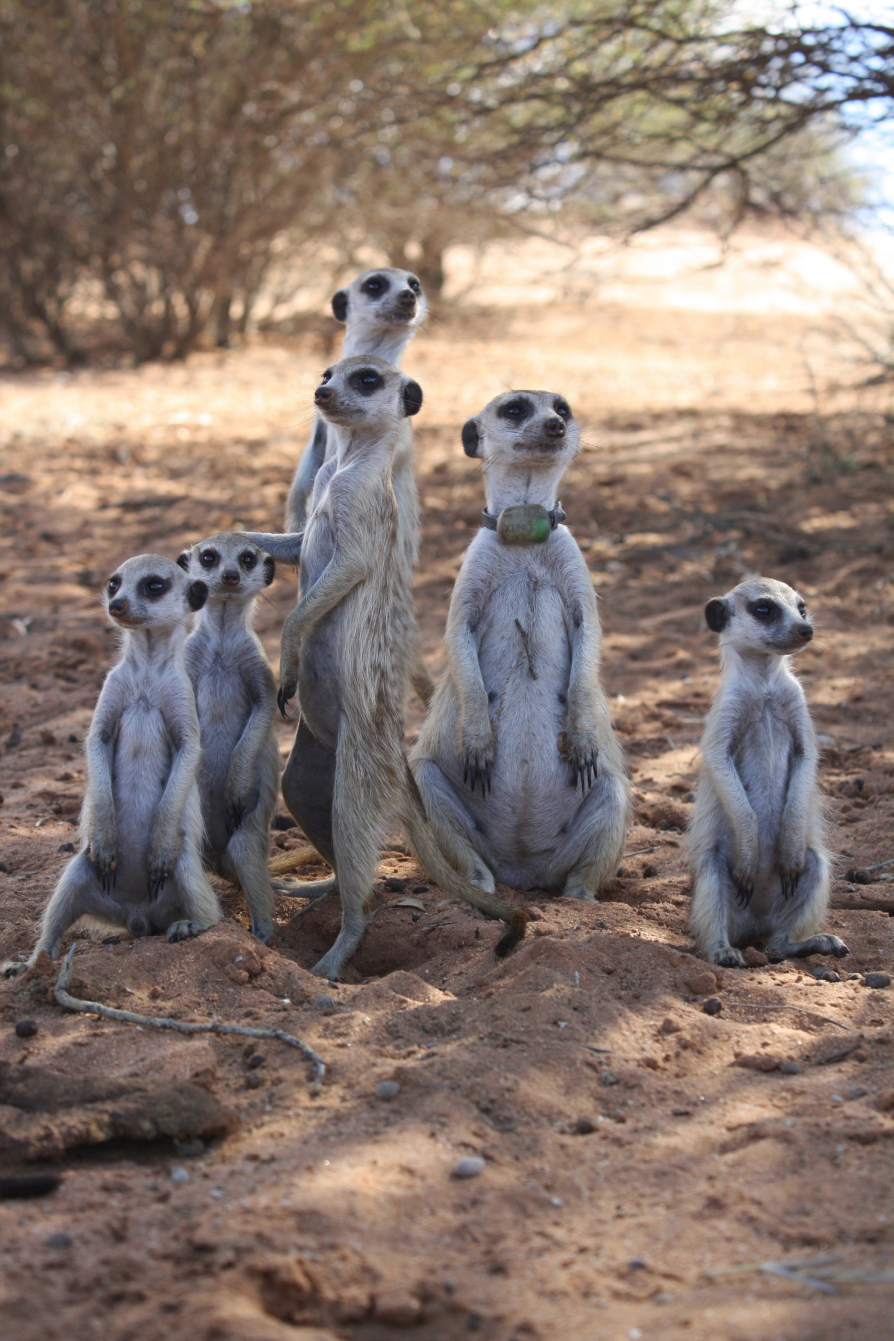
Members of a meerkat (*Suricata suricatta*) clan in the Kurman River Reserve, South Africa. The pregnant, dominant female is shown wearing a radio collar. Photo courtesy of Lydia K. Greene.

Maternal effects, whereby a mother's phenotype or internal environment shapes her developing offspring's phenotype, are ubiquitous, occurring via non‐genetic mechanisms involving the transfer of nutrients, antibodies and hormones (Mousseau and Fox 1998). Health effects on offspring owing to the maternal endocrine environment have been predominantly studied in birds and egg‐laying reptiles, in which exposure to maternal yolk androgens can alter offspring susceptibility to parasites (Tschirren, Richner, and Schwabl 2004) and immune function (reviewed in Groothuis, Müller, et al. 2005). Owing to the more prolonged and intimate physiological associations within maternal‐foetal units, maternal endocrine effects should be particularly pronounced in viviparous species. Indeed, stress‐induced increases in maternal glucocorticoids can impact offspring health and development (Welberg and Seckl 2001), producing stronger and potentially more damaging or even maladaptive effects in viviparous than oviparous species (MacLeod, While, and Uller 2021). To our knowledge, however, the health costs of natural maternal androgens on the offspring of placental mammals have yet to be explored in situ. Here, we test for such costs in a wild population of meerkats (*Suricata suricatta*) (Figure 1). In this cooperatively breeding carnivoran, maternal androgen concentrations are exceptionally high both relative to male peers and to other female mammals but vary significantly by the dams' social status (Davies et al. 2016), differentially influencing offspring behavioural development (Drea et al. 2021). We thus examine intergenerational costs associated with normative or experimentally induced maternal hormone variation (i.e. differential maternal androgen exposure or blockade, respectively) on parasitism, immune function and survivorship in juvenile and adult offspring.

In oviparous vertebrates, manipulation of yolk hormones reveals multiple downstream effects of maternally derived androgens on immune function (Groothuis, Müller, et al. 2005): Relative to controls, hatchlings from androgen‐treated eggs generally benefit from increased growth rates (Eising et al. 2001; Uller and Olsson 2003; Groothuis, Müller, et al. 2005) at the cost of increased susceptibility to parasites (Uller and Olsson 2003; but see Navara, Hill, and Mendonça 2006; Cucco et al. 2008; Muriel et al. 2015) and reduced cell‐mediated and humoral immunity (Groothuis, Müller, et al. 2005; Müller, Groothius, Kasprzik, et al. 2005; Sandell, Tobler, and Hasselquist 2009; Clairardin et al. 2011). Speculation that early effects of androgens on the immune system may be long‐lasting led some to view such effects as ‘organisational’ (Klein 2000; Tobler et al. 2010; Hasselquist, Tobler, and Nilsson 2012), a term traditionally reserved for permanent hormonal effects on sexual differentiation (Arnold and Breedlove 1985). There is currently no evidence, however, that health costs of embryonic androgen exposure persist in sexually mature birds (Cucco et al. 2008; Tobler et al. 2010; Bonisoli‐Alquati et al. 2011; Ruuskanen et al. 2013); reversible effects suggest the immature immune system can overcome initial deficiencies. We therefore should refer instead to ‘endocrine‐immune interactions’ when considering these sorts of developmental trade‐offs.

Our understanding of endocrine‐parasite or endocrine‐immune trade‐offs in mammals derives primarily from studies examining the concurrent or activational hormonal status of adults and, outside of the rare inclusion of ‘androgenized’ females (Smyth and Drea 2016; Smyth et al. 2016, 2018; Zohdy et al. 2017), typically reflect the traditional male bias in androgen concentrations (e.g. Schalk and Forbes 1997, but see Morales‐Montor et al. 2004; Klein 2000). We expect that trade‐offs in offspring development and health mediated by prenatal androgen exposure also operate in mammals. In several female‐dominant species, including meerkats, exposure to maternal androgens, particularly androstenedione (A_4_) and testosterone (T), impacts offspring morphology, physiology, behaviour and reproduction (Conley et al. 2020; Drea et al. 1998, 2021; Dloniak, French, and Holekamp 2006; Grebe et al. 2019), but we know little about its effects on offspring health. Perhaps the benefits from enhanced exposure to maternal androgens, experienced as enhanced competitive ability (Dloniak, French, and Holekamp 2006; Drea et al. 2021; Drea and Davies 2022) and early offspring growth (Hodge et al. 2008; Davies et al. 2024), are balanced by associated immunosuppressive effects.

We tested this proposition in the meerkat—a female‐led, social mongoose that lives in established clans comprising a dominant pair and multiple subordinate helpers of both sexes. Relative to male conspecifics, all female meerkats have exceptionally high A_4_ and T concentrations, with the dominant female per clan (the matriarch), whilst pregnant, having the greatest androgen concentrations of all (Davies et al. 2016). Androgen‐mediated competitiveness (Drea et al. 2021) enables her to largely monopolise reproduction (Griffin et al. 2003; Clutton‐Brock et al. 2006). Although subordinate females are physically capable of breeding and do so opportunistically (Clutton‐Brock et al. 2001; Dimac‐Stohl et al. 2018), their reproductive success is often thwarted behaviourally by inbreeding‐ avoidance tactics, infanticide, female eviction or litter abandonment (Clutton‐Brock et al. 1998, 2006; Young et al. 2006; Drea and Davies 2022). Despite gaining enormous reproductive success, the matriarch also incurs androgen‐related costs, including increased gastrointestinal parasite burdens (Smyth et al. 2016) and reduced innate immune function (Smyth et al. 2018). Given that androgens in adult female meerkats are immunosuppressive and reach developing foetuses during pregnancy (Drea et al. 2021), the androgen‐immunocompetence trade‐off (collectively referring to parasitism and innate immune function) could extend to the matriarch's offspring. Accordingly, any advantage accrued to her pups might be offset by a health handicap.

Capitalising on natural, status‐related variation in androgen concentrations in pregnant meerkats residing within the clan (Davies et al. 2016)—a differential pattern not observed in glucocorticoids (Dimac‐Stohl et al. 2018)—we first looked for a potential relationship between varying prenatal androgen exposure and immunocompetence in the offspring of dominant control (DC) and subordinate control (SC) dams. Second, capitalising on an experimental manipulation involving the administration of flutamide (an androgen‐receptor blocker) to dominant dams (Drea et al. 2021), we also examined offspring from these dominant treated (DT) individuals. Treatment was administered during the third trimester of the meerkat's 70‐day gestation because this period (1) coincides with a natural increase in gestational androgens, particularly in matriarchs (Drea et al. 2021), (2) is when the neural substrates underlying sexually differentiated behaviour in developing offspring respond to masculinising effects of androgens (reviewed in Wallen 2005) and (3) is when foetal bone marrow is established as the primary haematopoietic site (Dietert et al. 2000; Landreth 2002). Disrupting the development of the bone marrow microenvironment could alter the production of white blood cells.

Using three categories of offspring, from normative (DC and SC) and experimental (DT) dams, we tested the ‘trade‐off’ hypothesis that advantages of prenatal androgens are offset by suppressive effects on immunocompetence. We predicted the following: (1) ‘DC’ offspring, naturally exposed to the greatest maternal androgen concentrations prenatally, would experience increased parasitism and reduced immune function relative to ‘SC’ offspring; (2) blocking prenatal androgen action would prevent these costs, such that ‘DT’ offspring would harbour fewer parasites and show more robust immune responses relative to ‘DC’ offspring; (3) preventing these costs might improve offspring survival relative to control peers; and (4) based on the temporary immunosuppressive costs of yolk androgen exposure (Cucco et al. 2008; Bonisoli‐Alquati et al. 2011; Ruuskanen et al. 2013), the effects of placental androgen exposure would be stronger in juveniles than in adults. In adult meerkats, concurrent or activational androgen concentrations vary widely between individuals and relate negatively to parasitism and immune function (Smyth et al. 2016, 2018); however, given the immature reproductive endocrine system of juveniles (Davies et al. 2024), we did not expect concurrent androgen concentrations to similarly relate to immune function.

## 2 Materials and Methods

### 2.1 Study Site and Subjects

This study (spanning January 2013–March 2015) involved a well‐habituated, wild meerkat population in the Kuruman River Reserve (26°58′ S, 21°49′ E) in South Africa's Kalahari Desert (Figure 1). Individuals are microchipped and identifiable by unique dye marks; researchers can observe the animals at close range (< 1 m) and routinely collect life history and weight data. Details about the study site, habitat, climate and monitoring procedures have been provided elsewhere (Clutton‐Brock et al. 1999; Drea et al. 2021). All protocols were first approved locally by the Kalahari Research Trust, a not‐for‐profit organisation registered in South Africa that promotes research on the biology and conservation of the fauna of the Kalahari, then internationally by the University of Pretoria ethics committee (Ethical Approval Numbers EC074‐11 and EC080‐14) and the Duke University Institutional Animal Care and Use Committee (Protocol Registry Numbers A171‐09‐06 and A143‐12‐05).

Our subjects, initially, were 128 meerkat offspring (62 male, 66 female) from 24 clans, deriving from 51 DC, 20 SC and 5 DT litters (see maternal treatment procedures below). Respectively, they included 78 ‘DC’, 37 ‘SC’ and 13 ‘DT’ offspring in relatively equal sex ratios (Table 1). We studied these offspring at two life stages: juvenility (3–12 months) and adulthood (12–62 months) (Table 1; see Table 2 for mean ages and weights at sampling). Owing to the logistics of fieldwork, subjects differentially contributed to the biological samples (faeces and blood, see below); owing to the natural mortality or dispersal of subjects, we could sample only 55% of our juveniles again as adults. We thus supplemented our adult stage, as feasible, by sampling from among an additional 67 meerkats from the general population deriving from normative DC and SC dams. Our total subjects across both life stages thus represent 195 offspring (see Tables 1 and 2 for final sample sizes per age group or analysis).

**TABLE 1:** Scales of inference and numbers of replicates.

Scale of inference	Scale at which the factor of interest is applied	Number of replicates at the appropriate scale^a^
Starting offspring population	Maternal androgen exposure:	Litters/individuals (males, females):
	Dominant Control	51/78 (36 M, 42 F)
	Subordinate Control	20/37 (19 M, 18 F)
	Dominant Treated	5/13 (7 M, 6 F)
Life stage	Juvenility:	Litters/individuals/biological samples:
	Parasitism^b^	27/45/89
	Immunity^c^	21/46/46
	Adulthood^d^:	
	Parasitism^b^	48/71/109
	Immunity^c^	33/57/70

*Note:* ^a^For additional breakdown of replicate numbers, see Table 2. Biological samples are ^b^faeces or ^c^serum. ^d^Adulthood required an additional 67 subjects.

**TABLE 2:** Summary of parasitism (parasite species richness, PSR; faecal egg counts, FEC) and immune function (bacteria killing ability, BKA scores; haemolytic complement activity, HCA scores) in juvenile and adult meerkat offspring, by maternal category; age and weight at time of sampling are also provided.

Offspring measure	Total numbers^a^ and mean (SE) values by maternal category		
	Dominant control	Subordinate control	Dominant treated
Juvenile parasitism			
Replicates^b^	12/17/32	11/19/29	4/ 9/28
Age (months)	8.46 (0.49)	8.63 (0.53)	8.72 (0.43)
Weight (g)	529.63 (20.50)	451.47 (22.21)	512.54 (12.03)
PSR	2.84 (0.19)	2.76 (0.23)	1.79 (0.20)
FEC	334.93 (113.23)	112.94 (51.59)	43.89 (14.80)
Juvenile immunity			
Replicates^c^	7/16/16	12/23/23	2/7/7
Age (months)	3	3	3
Weight (g)	314.25 (16.42)	302.46 (15.73)	342.86 (15.80)
BKA (% killed)	57.79 (5.98)	45.30 (4.84)	83.70 (8.49)
HCA (CH_50_)	165.46 (17.87)	158.36 (8.02)	—
Adult parasitism			
Replicates^b^	36/53/75	8/12/19	4/6/15
Age (months)	24.23 (1.27)	19.67 (630.03)	18.46 (1.25)
Weight (g)	618.08 (12.89)	630.03 (29.28)	582.80 (20.66)
PSR	2.43 (0.13)	2.11 (0.24)	1.87 (0.39)
FEC	183.31 (47.60)	55.57 (16.82)	109.00 (46.59)
Adult immunity			
Replicates^c^	20/34/40	8/13/17	5/10/13
Age (months)	25.15 (2.75)	20.52 (2.68)	20.84 (2.51)
Weight (g)	638.98 (13.02)	626.69 (21.24)	655.65 (25.53)
BKA (% killed)	52.57 (3.40)	47.65 (5.68)	51.80 (7.36)
HCA (CH_50_)	229.64 (6.02)	207.30 (12.13)	205.63 (12.06)

*Note:* ^a^Total replicate numbers represent litters/individual meerkats/biological samples. Biological samples are ^b^faeces or ^c^serum.

In meerkat society, all offspring are subordinate, regardless of maternal status. Among adult subordinates, there is some status differentiation, largely based on age or size advantage (Thavarajah, Fenkes, and Clutton‐Brock 2014), with the eldest/largest being most likely to emerge as dominant in individual contests that result in an overthrow (Clutton‐Brock et al. 2006; Hodge et al. 2008). A few of our adult offspring became dominant: To avoid the possible confounding effects of social status on immunocompetence (Smyth and Drea 2016; Smyth et al. 2016, 2018), we stopped sampling animals upon the onset of dominance disputes or acquisition of breeding status.

### 2.2 Experimental Treatment

The androgen‐receptor antagonist Flutamide had been experimentally administered during 11 pregnancies in dominant dams, five of which either bore offspring that survived to emergence from the natal den or continued to reside in the study population during key periods (Drea et al. 2021). Prior to the experimental manipulation, each dam's health, weight gain and abdominal size throughout pregnancy had been closely monitored (Dimac‐Stohl et al. 2018). The dams had been selected to match by age and clan size. Early in the third trimester, candidates identified for treatment had been subcutaneously implanted with ~15 mg/kg/day flutamide (2 × 21‐day release, 150‐mg pellets, Innovative Research of America, Sarasota, FL), targeting the last 21 days of the 70‐day gestation period. The capture, anaesthesia, blood draw, surgical and post‐procedural monitoring methods are published (Drea et al. 2021). For each DT dam, we followed both her treated litter and one of her control litters, implementing a within‐subject crossover design. To comply with a request by local stakeholders at the Kuruman River Reserve, we kept invasive procedures of these key dominant females to a minimum. A prior validation of our procedures was conducted on subordinate males, indicating no difference between ‘handling’ controls (i.e. those captured, anaesthetised and sampled) and ‘surgical’ controls (i.e. those additionally implanted with a placebo pellet) (delBarco‐Trillo et al. 2016). Our study of dominant females thus involved ‘handling’ controls only (Drea et al. 2021).

### 2.3 Sampling Procedures

To examine parasitism, we opportunistically collected fresh faeces from juvenile (3–11 months) and adult (12–51 months) offspring. Faecal collection, storage, transport and processing methods followed Smyth and Drea (2016).

To examine immune function, as well as the relationships between concurrent sex hormone concentrations and immune function, we collected serum from offspring as juveniles (at 3 months) and adults (over a range spanning 12–62 months). We followed published procedures for animal capture, blood collection and serum processing (Davies et al. 2016). Because the innate immune components in serum are unaffected by long‐term storage (up to 12 months) at −80°C (Smyth et al. 2018), we kept serum at this temperature throughout all phases of storage and transport, until analysis. Due to age‐related constraints on serum volume, our sample sizes varied per immune measure (Table 2).

### 2.4 Measures of Parasitism

Our meerkat population is infected by six endoparasite taxa—four nematodes (Strongylates, *Toxocara suricattae*, Spiurids and *Oxynema suricattae*), one cestode (*Pseudandrya suricattae*) and one apicomplexan (coccidia) (Leclaire and Faulkner 2014; Smyth and Drea 2016). From each faecal sample, we recovered endoparasite eggs from 4.5 g of material by water wash and centrifugal flotation in 11 mL Sheather's solution (Smyth and Drea 2016). We quantified the number of eggs or oocysts (hereafter ‘eggs’) per sample and used parasite species richness (PSR or the number of parasite taxa present) and faecal egg counts (FEC) as indices of infection. FEC represents an estimate of total infection intensity. Although egg output can vary considerably between parasite taxa (Gillespie 2006), FEC has been shown to linearly relate to adult parasite burdens (Roberts and Swan 1981; Seivwright et al. 2004). We refer to PSR and FEC, collectively, as our measures of parasitism.

### 2.5 Measures of Innate Immune Function

We assessed constitutive, innate immune responses using a bacteria‐killing assay (BKA) and haemolytic complement assay (HCA), following Smyth et al. (2018). The BKA and HCA are functional tests of the innate immune system's ability to control bacteria and lyse foreign antigens, respectively, and involve the action of two interrelated immune components—natural antibodies and complement. Because these measures have been linked to disease resistance and survival in free‐ranging animals (Townsend et al. 2010; Wilcoxen, Boughton, and Schoech 2010; Savage et al. 2016), researchers can use assay results to make health inferences.

We optimised the BKA and HCA for meerkats, as described previously (Smyth et al. 2018), by modifying protocols developed by French and Neuman‐Lee (2012) and Sinclair and Lochmiller (2000), respectively. We represent BKA scores as the percentage of bacteria killed relative to positive controls and HCA scores in CH_50_ units (i.e. the reciprocal of the dilution that causes 50% haemolysis) (Mayer 1948). We refer to BKA and HCA scores, collectively, as our measures of immune function.

### 2.6 Hormone Assays

We assayed serum samples for A_4_, T and oestradiol (E_2_) using commercial, competitive enzyme immunoassay kits (ALPCO Diagnostics, Salem, NH, USA), previously validated in meerkats (Davies et al. 2016). We ran all samples in duplicate, and if the coefficient of variation (CV) exceeded 10%, we ran a subsequent assay. The A_4_ assay has a sensitivity of 0.04 ng/mL using a 25‐μL dose, with intra‐ and inter‐assay CVs of 5.23% and 8.7%, respectively. The T assay has a sensitivity of 0.02 ng/mL using a 50‐μL dose, with intra‐ and inter‐assay CVs of 7.9% and 7.3%, respectively. The E_2_ assay has a sensitivity of 10 pg/mL using a 50‐μL dose, with intra‐ and inter‐assay CVs of 7.7% and 8.7%, respectively.

### 2.7 Statistical Analyses

In juveniles, we tested for differences in parasitism (PSR and FEC) and immune function (BKA and HCA scores) using linear models (LMs) and linear mixed models (LMMs). We included the following explanatory variables in all models: maternal category (three levels: DC, S, and DT), weight (continuous variable in g) and their interaction. For individuals sampled over a range of ages, we controlled for age (continuous variable in mo) by including it as an explanatory factor. Because rainfall is negatively associated with endoparasitism in meerkats (Smyth and Drea 2016), we also controlled for rainfall (continuous variable in mm, summed over 30 days pre sampling) by including it in our analyses of PSR and FEC. A small sample size for certain status‐by‐sex combinations meant that we lacked the statistical power to include sex in the analyses of juveniles. We repeated the prior analyses in adult offspring, for which we could include sex (two levels: male, female) as an additional explanatory factor.

To test if immune function in juveniles is related to concurrent sex hormone concentrations, we examined relationships between our two immune responses (BKA and HCA scores) and three sex steroids (A_4_, T and E_2_, each as a continuous variable in ng/ml) using LMs. We did not perform these analyses in adults because relationships between normative immune function and sex steroid concentrations have been reported for adults (Smyth et al. 2018) and serum volumes from treated dams were limited.

For all models, we confirmed that the necessary assumptions had been met by visually inspecting quantile‐quantile plots and plots of standardised residuals versus fitted values. We log‐transformed adult BKA scores (residuals for juvenile BKA scores were normally distributed) and FECs (adding 1 to each value prior to transformation). We centred and scaled all continuous variables by subtracting the mean and dividing by the standard deviation. To assess collinearity between all explanatory factors, we used variance inflation factors (VIFs); we retained all variables because all VIFs were under 2 (O'Brien 2007). We controlled for repeated sampling of individuals by including as random terms either individual identity or individual identity nested within clan identity. We determined the optimal random structure for each model by estimating variance components.

We evaluated the significance of fixed effects using *F* statistics (for LMs) and likelihood ratio tests (for LMMs); we present effect sizes (means and standard errors) for all fixed effects. If maternal category was a significant predictor (*α* < 0.05), we used least‐squares means and Dunnett's multiple comparison tests to examine differences between DC and SC progeny (the normative comparison) and between DC and DT progeny (the experimental comparison). Owing to age‐related differences in serum volume, we were unable to perform experimental comparisons for HCA values in juveniles.

We used a Cox proportional hazards model to test for effects of maternal status or treatment condition on offspring survivorship, used a likelihood ratio test to determine the significance of maternal status and verified that the proportional hazards were equal among individuals. We calculated pairwise comparisons among treatments using a Bonferroni correction for multiple comparisons. We performed all statistical analyses in Program R version 4.2.2 (R Core Team 2022). We used the *nlme* package (Pinheiro et al. 2022) for fitting LMMs, the *lsmeans* package (Lenth 2016) for comparisons of least‐squares means and the *survival* (Therneau 2022) and *survminer* (Kassambara, Kosinski, and Biecek 2021) packages for survival analyses.

## 3 Results

### 3.1 Offspring Parasitism by Maternal Category

**FIGURE 2:**
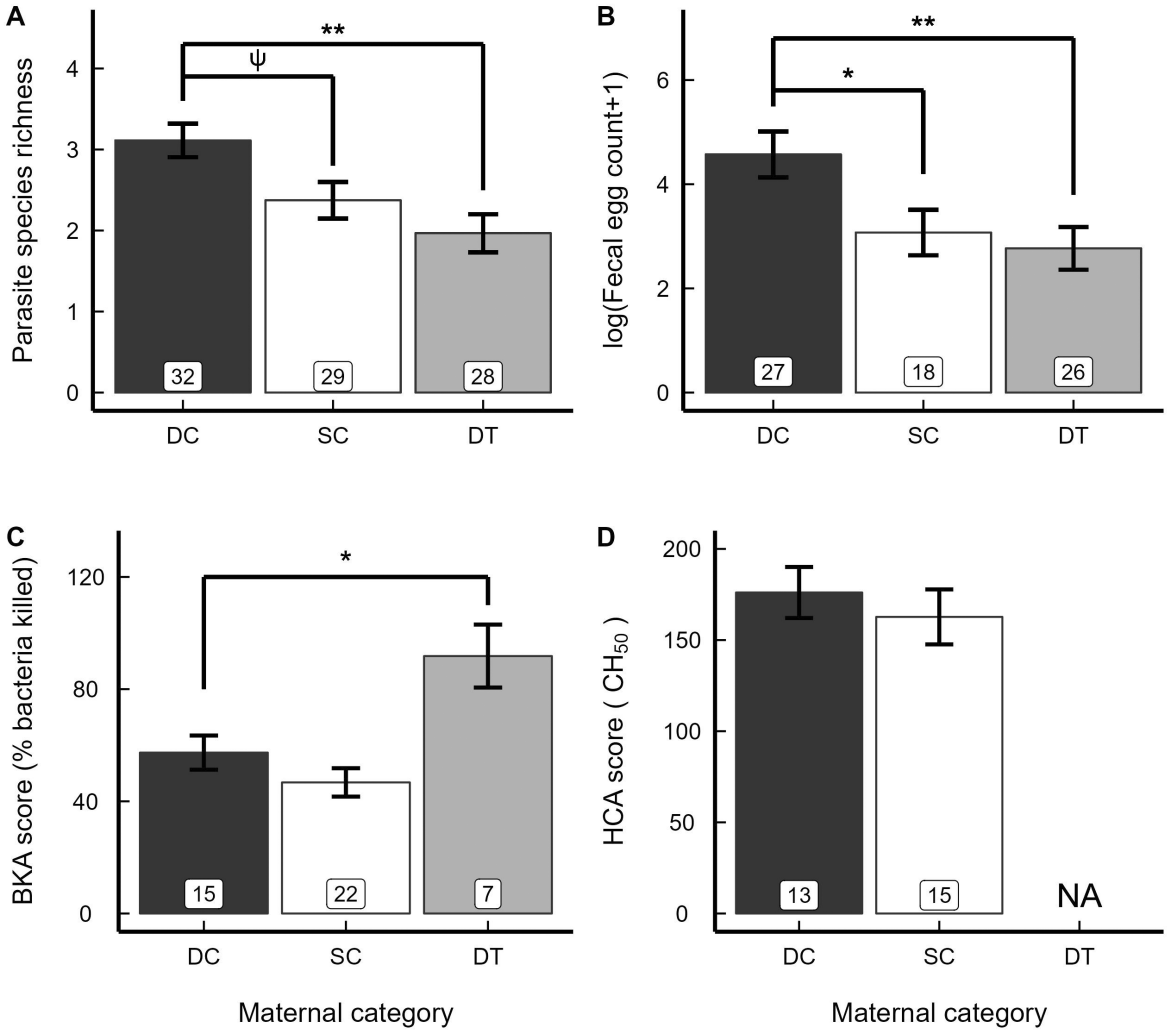
Predicted means ± SEM (from model statistics) of (A) endoparasite species richness, (B) endoparasite faecal egg count, (C) bacteria killing ability (BKA score) and (D) haemolytic complement activity (HCA score) in juvenile meerkat offspring by maternal category (dominant control (DC), black; subordinate control (SC), white; dominant treated (DT), grey). Shown are results from the a priori, ‘normative’ comparisons between juveniles from DC and SC dams and ‘experimental’ comparisons between juveniles from DC and DT dams. Because we made no a priori predictions for SC vs. DT females, there was no test of that comparison. Boxed numbers represent sample sizes. **p < 0.01, *p < 0.05, ^ψ^ *p* < 0.10.

Maternal effects on parasitism in juvenile offspring followed predicted patterns. First, the status‐related variation in androgen concentrations of normative dams was positively related to parasitism in juvenile offspring: relative to ‘DC’ offspring, ‘SC’ offspring tended to have reduced PSR (*p* = 0.058, n.s.) and had significantly reduced intensities of parasite infections, as indicated by FECs (*p* = 0.031) (Table 2; Figure 2A,B; Table A1). Second, variation in androgen action owing to treatment of dominant dams also influenced offspring parasitism: relative to ‘DC’ offspring, ‘DT’ offspring had significantly reduced parasite burdens (PSR: *p* = 0.003; FEC: *p* = 0.004) (Table 2; Figure 2A,B; Table A1). Indeed, parasitism in the juvenile progeny of DT dams was comparable to that in the juvenile progeny of SC dams (Table 2; Figure 2A,B).

**FIGURE 3:**
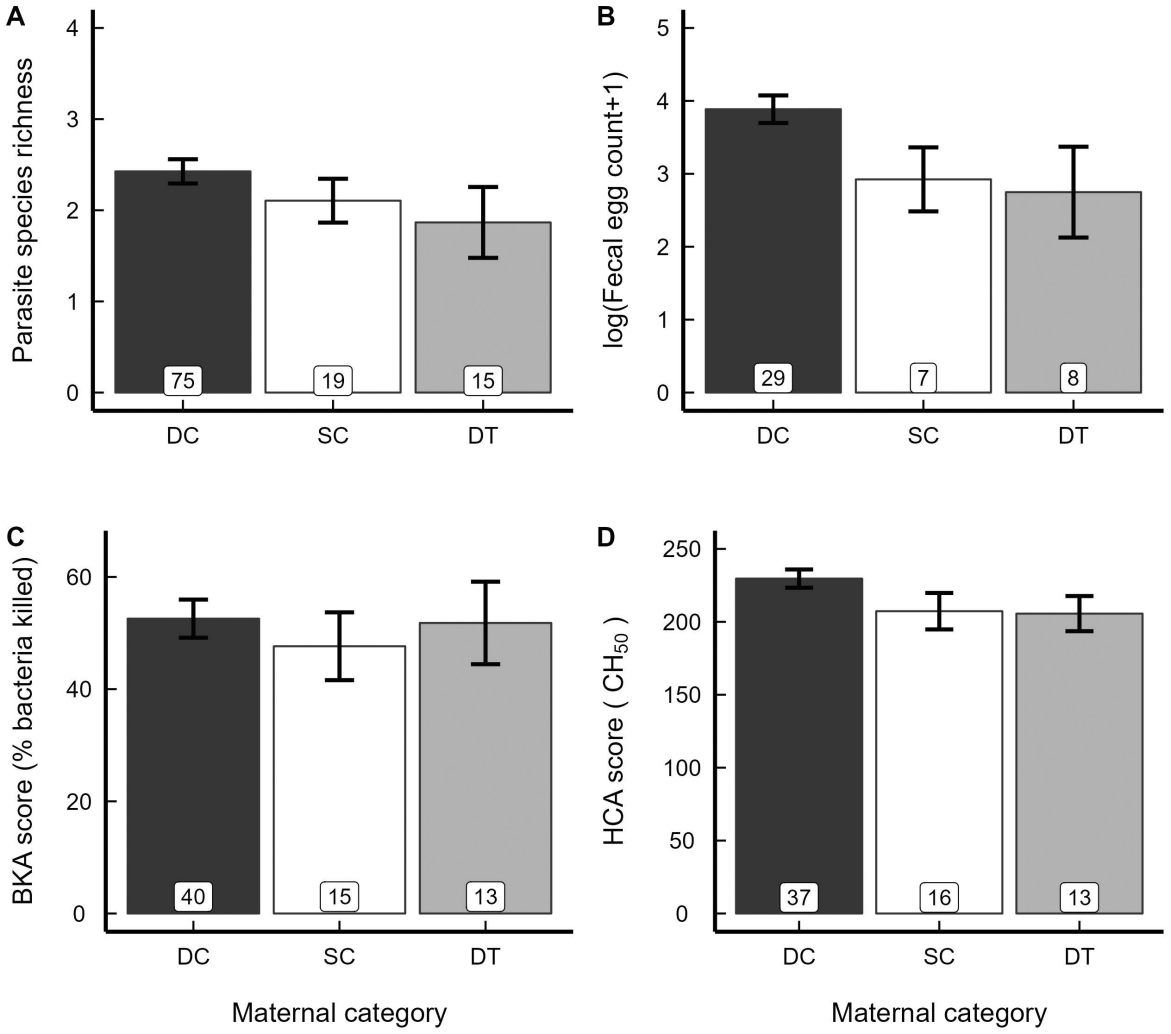
Means ± SEM (from raw data) of (A) endoparasite species richness, (B) endoparasite faecal egg count, (C) bacteria killing ability (BKA score) and (D) haemolytic complement activity (HCA score) in adult meerkat offspring by maternal category (dominant control (DC), black; subordinate control (SC), white; dominant treated (DT), grey). None of the a priori ‘normative’ comparisons between adult offspring from DC and SC dams or ‘experimental’ comparisons between adult offspring from DC and DT dams were statistically significant. Boxed numbers represent sample sizes.

Consistent with findings in other taxa, these maternal effects did not persist over time. Once the offspring of normative and treated dams reached sexual maturity, none of these maternal effects remained detectable (all *p*s > 0.05) (Table 2; Figure 3A,B; Table A2).

### 3.2 Offspring Immune Function by Maternal Category

Our findings relating to maternal effects on immune function in juvenile offspring showed both a null effect and an effect consistent with predictions. The normative status‐related variation in maternal androgen concentrations did not generate detectable differences in offspring immune function, as the progeny of DC and SC dams had comparable BKA and HCA scores (*p*s > 0.05) (Table 2; Figure 2C,D; Table A1). By contrast, experimentally blocking androgen action in DT dams had anticipated consequences in that, relative to ‘DC’ juveniles, ‘DT’ juveniles had significantly improved immune responses (BKA scores: *p* = 0.020; HCA scores were unavailable for ‘DT’ juveniles) (Table 2; Figure 2C,D; Table A1).

As with parasitism, no androgen‐related maternal effects on immune function (for either BKA or HCA scores) were apparent in the adult offspring of DC, SC and DT dams (all *p*s > 0.05) (Table 2; Figure 3C,D; Table A2).

### 3.3 Other Predictors of Parasitism and Immune Function: Age, Weight, Reproductive Hormones and Sex

**FIGURE 4:**
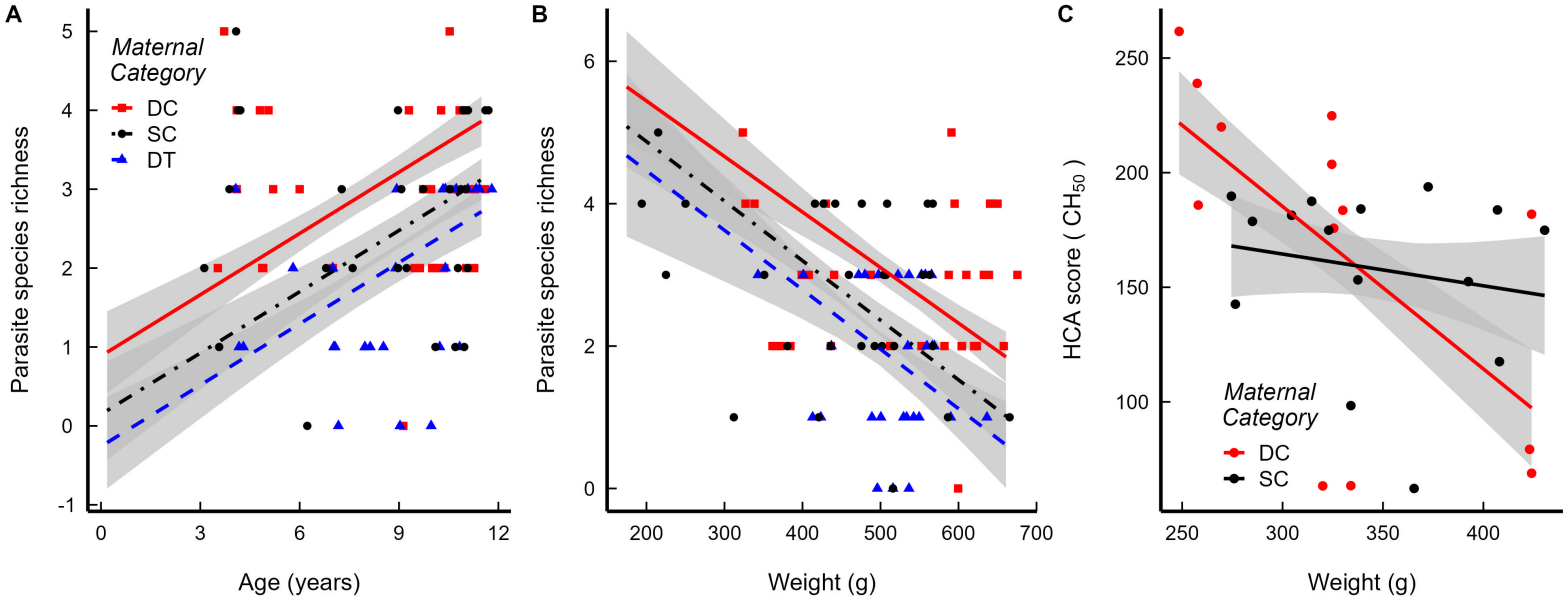
Demographic predictors that significantly explain variance in (A, B) endoparasite species richness and (C) haemolytic complement activity (HCA scores) in juvenile meerkat offspring (Table A1). Dots represent individual samples (dominant control (DC), red; subordinate control (SC), black; dominant treated (DT), blue). Lines represent the predicted fits of a linear mixed model (A, B) and a linear model (C) for each maternal category available. Shaded ribbons indicate standard error estimates.

**FIGURE A1:**
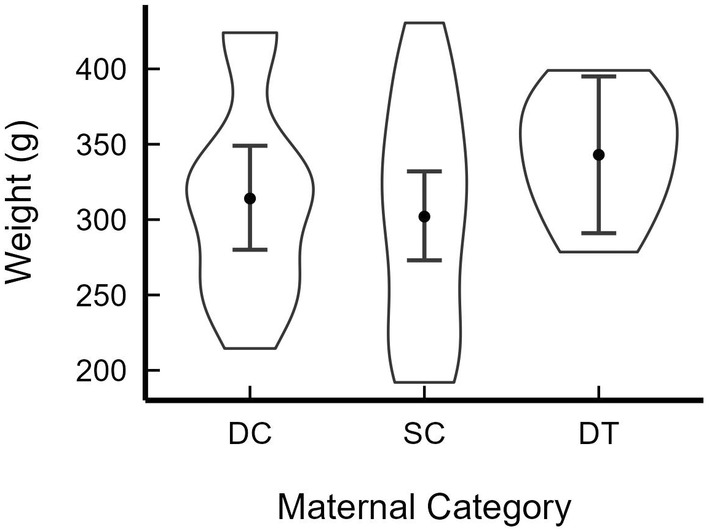
Violin plots showing mean (± 95% confidence intervals) body weight (g) of juvenile meerkats at 3 months of age by the status or treatment group of their mothers, including dominant control (DC), subordinate control (SC) and dominant treated (DT) dams that received an androgen‐receptor blocker in late gestation. Weight did not differ among treatment groups (DT‐DC *t* ratio = 0.927, *p* = 0.359; DT‐SC *t* ratio = 1.37, *p* = 0.179). Violins represent the distribution of individuals; red lines represent the upper and lower 95% confidence intervals as determined by a linear model.

**FIGURE A2:**
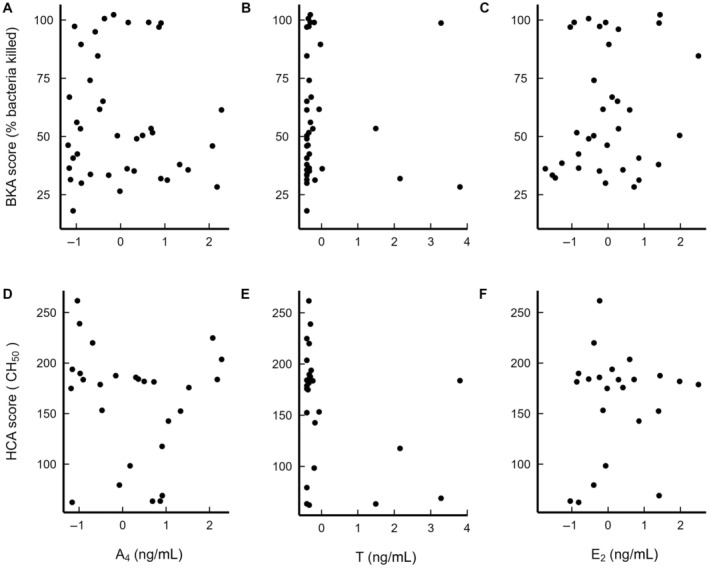
Concurrent sex hormone (androstenedione, A_4_; testosterone, T; oestradiol, E_2_) concentrations are unrelated to innate immune function in juvenile meerkat offspring. Bacteria killing ability (BKA scores, row 1) and haemolytic complement activity (HCA scores, row 2) are plotted against A_4_ (column 1), T (column 2) and E_2_ (column 3). Statistics are provided in Table A3.

Juvenile age predicted PSR, independent of maternal category: older juveniles were infected with more species of endoparasites than were younger juveniles (Figure 4A; Table A1). Juvenile body weight, which was unrelated to the maternal category in our study (*p* > 0.05) (Table A1; Figure A1), predicted both PSR and HCA scores: Relative to their lighter counterparts, heavier juveniles were infected with fewer species of gastrointestinal parasites (*p* = 0.003), suggesting enhanced immunocompetence, but also had reduced HCA scores (*p* = 0.011), suggesting reduced immunocompetence (Figure 4B,C; Table A1). Lastly, the concurrent concentrations of A_4_, T and E_2_ in 3‐month‐old juveniles were unrelated to maternal status or to BKA and HCA scores (all *p*s > 0.05) (Table A3; Figure A2).

As before, the age and weight relationships evident in juvenility were not detectable in adult offspring (Table A2). Instead, sex was the only factor that significantly related to immune function in older offspring, with females having greater BKA scores than males (*p* = 0.03) (Table A2).

### 3.4 Survivorship

**FIGURE 5:**
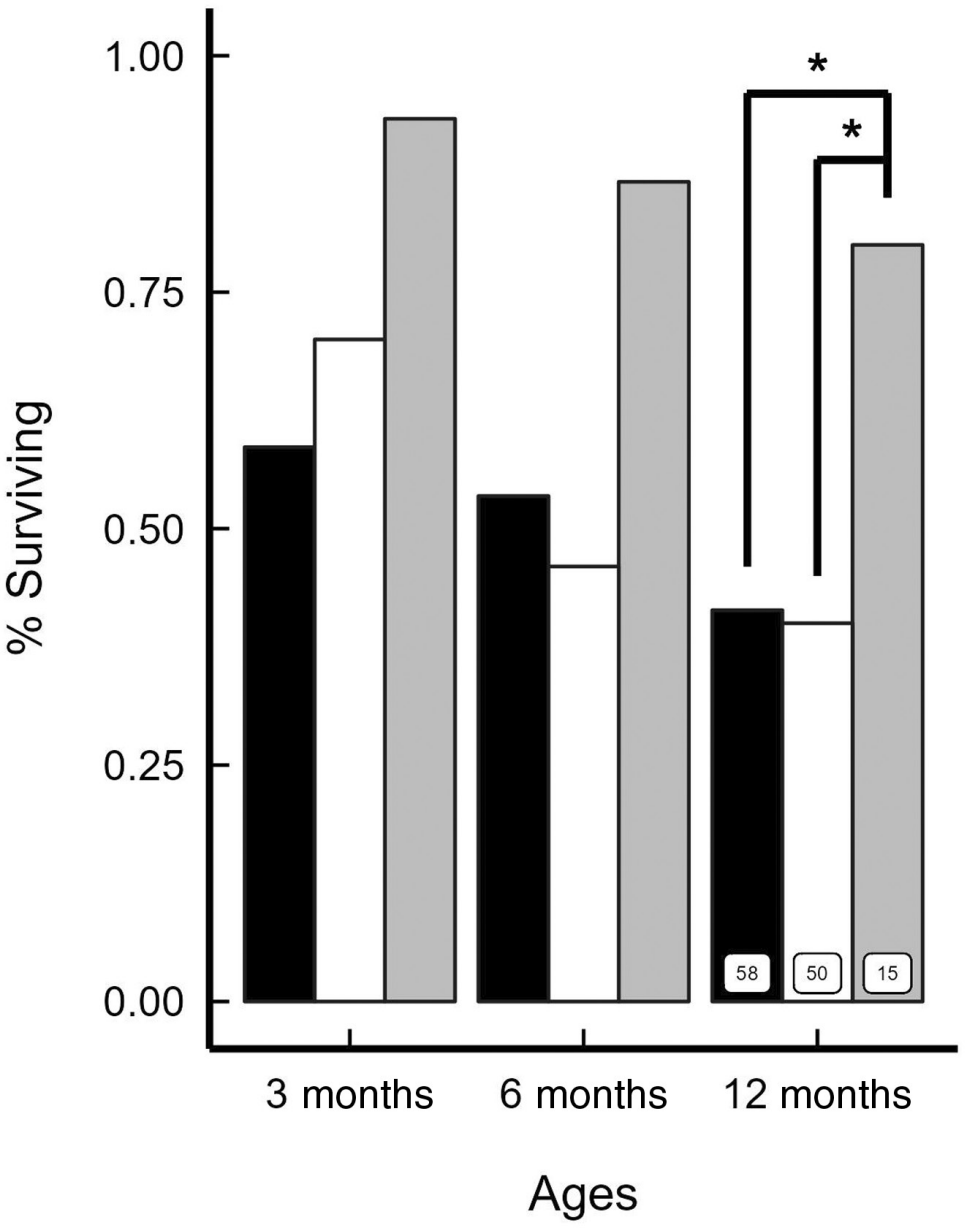
Survivorship of meerkat offspring in relation to maternal categories (dominant control (DC), black; subordinate control (SC), white; dominant treated (DT), grey). Shown at 12 months are comparisons between juveniles from DC and DT dams, and between juveniles from SC and DT dams, with offspring numbers (in boxes). *p < 0.05.

Offspring survivorship varied with maternal category (*χ* ^2^ = 9.51; df = 2; *p* = 0.008). We found no difference between ‘DC’ and ‘SC’ offspring (*p* = 1.000), whereas ‘DT’ offspring had significantly greater survival than either ‘DC’ (*p* = 0.030) or ‘SC’ (*p* = 0.024) offspring (Figure 5).

## 4 Discussion

The immunocompetence handicap hypothesis (ICHH) (Folstad and Karter 1992), originally positing an androgen‐mediated trade‐off between reproductive benefits and immunosuppression in adult males, was recently extended to adult female meerkats (Smyth et al. 2016, 2018). Here, for the first time in a wild mammal, we report that prenatal exposure to maternal androgens also has immunosuppressive effects on juvenile offspring of both sexes: Relative to the progeny of DC dams exposed to the highest concentrations of maternal androgens in utero, the progeny of SC and DT dams experienced enhanced immunocompetence (as revealed by any combination of PSR, FEC, BKA or HCA). The health benefits of blocking late‐term, prenatal androgen action in dominant dams significantly improved survivorship of their progeny (i.e. DT offspring) relative to offspring that had prenatally experienced a range of normative maternal androgen concentrations, be they exceptionally or moderately raised (i.e. in DC or SC offspring, respectively). These findings validate an earlier report of a trend towards better survivorship in DT relative to DC offspring, based on smaller sample sizes of normative offspring examined only to 1 year of age (Davies et al. 2024). Thus, despite the reproductive benefits of hormonal masculinisation to dominant female meerkats (Drea et al. 2021), the intergenerational consequences of raised maternal androgens on immune function and survivorship are naturally experienced by their juvenile offspring and appear to represent an early cost of sexual selection (see Clutton‐Brock 2001) operating in fiercely competitive females.

We interpret these costs as owing to maternal androgens for several reasons. Firstly, that health effects across all offspring were restricted to the juvenile period, encompassing animals at 3 months of age that have circulating hormone concentrations lower than those in adulthood, implicate organisational rather than activational effects. Secondly, flutamide specifically blocks the androgen receptor, without modifying concentrations of androgens (delBarco‐Trillo et al. 2016; Drea et al. 2021) or their downstream oestrogenic products. Whereas oestrogen concentrations in normative meerkat dams also vary by status (reviewed in Drea and Davies 2022), potentially contributing to the differences observed between DC and SC progeny, androgen action more parsimoniously explains the full suite of findings. Notably, differences between DC and DT offspring can only be explained by maternal androgen action. Thirdly, the breadth of effects observed in DT versus SC dams is consistent with expectations from blocking the mechanism of androgen action versus merely lowering absolute concentrations. Lastly and more generally, our results are consistent with the literature on androgen manipulation in oviparous species.

Regarding immune function, offspring did not appear to suffer the status‐related trade‐off experienced by their mothers. Perhaps a threshold of prenatal androgen exposure exists, above which further increases in androgens no longer affect prenatal programming of innate immune function. Because subordinate dams are also hormonally masculinised, albeit to a lesser extent than dominant dams, differences in innate immune function between their respective offspring could be minimal. Detecting these differences would require greater statistical power, especially if the immunomodulatory effects are influenced by offspring sex (Müller, Groothuis, Eising, et al. 2005; Muriel et al. 2017) or litter sex ratios (via exposure to fraternal androgens: Ryan and Vandenbergh 2002).

Several mechanisms may explain relationships between prenatal androgens and immune function or parasitism. Androgens could bind to receptors in developing immunocytes (Kovacs and Olsen 1987), directly modifying these immune cells and altering their functions or future responses to androgenic stimulation. Androgens could also act on lymphoid organs during critical developmental periods, altering the micro‐environments in which immune cells mature throughout life (Martin 2000). Organisational effects of maternal androgens on the foetal central nervous system could, by enhancing offspring aggressiveness (Dloniak, French, and Holekamp 2006; Drea et al. 2021), boldness (Partecke and Schwabl 2008) or exploratory behaviour (Holekamp et al. 1984), indirectly increase exposure to parasites. Similar effects on the foetal endocrine system could alter future hormone production (Drea et al. 1998) or receptor sensitivity and distribution pattern (Carere and Balthazart 2007), including in lymphoid organs (Groothuis and Schwabl 2008). Although immune function was unrelated to concurrent concentrations of A_4_, T and E_2_ in 3‐month‐old juvenile meerkats, perhaps owing to endocrine‐system quiescence or immature hormone expression, previous findings in adults are suggestive of concurrent androgenic effects on parasitism (Smyth et al. 2016) and immune function (Smyth et al. 2018).

Beyond the mechanisms detailed above, prenatal androgens could alter immunocompetence via trade‐offs with other physiological processes (Sheldon and Verhulst 1996; Lochmiller and Deerenberg 2000; Demas 2004), including growth (Uller and Olsson 2003; Andersson et al. 2004; Groothuis, Eising, et al. 2005; Müller et al. 2010). Such trade‐offs, which arise from allocating finite resources to energetically expensive activities, may be most evident during periods of intense competition or reduced food availability (Uller and Olsson 2003; Andersson et al. 2004). Indeed, the inverse relationship between body weight and haemolytic complement activity in juvenile meerkats is concordant with a trade‐off between growth and immune function. That we observed increased parasitic infections in light‐weight individuals – a pattern opposite the norm – does not negate this trade‐off but could indicate nutritional deficiencies imposed by multiple concurrent infections (Sykes, Poppi, and Elliot 1988). Nevertheless, because meerkats are most vulnerable to predation and starvation during the first months of life (Clutton‐Brock et al. 1999), body mass and rapid growth may be more important than immunity for ensuring immediate survival. Indeed, early body weight and competitive growth are critical in meerkat society (English et al. 2013; Huchard et al. 2016). By increasing survival during this period, raised gestational androgens might benefit offspring growth (Drea and Davies 2022; Davies et al. 2024), especially if the immunosuppressive costs of raised androgens are short‐lived.

## 5 Conclusion

The trade‐offs associated with hormone transfer from mother to offspring in avian systems (Groothuis and Schwabl 2008) may not have translated to placental mammals because of the marked developmental differences between oviparous and viviparous species. In birds, maternal hormone deposition occurs just once around the time of oviposition, whereas in mammals, hormone transfer is more dynamic and can occur at any time for varying durations throughout gestation, with time‐sensitive consequences. In meerkats, maternal androgens naturally transfer to developing pups during the last third of gestation (Drea et al. 2021); our findings show that these androgens exert similar negative effects on the early immunocompetence of offspring as those observed in oviparous species. In female‐dominant mammals, such as the meerkat, there may be a conflict between the optimal endocrine environment for maximising the mother's competitiveness and, thus, lifetime reproductive success and the environment that best contributes to the early health of her developing offspring. Likewise, offspring may face their own developmental trade‐off between investing in growth versus maximising their health. That raised androgens during pregnancy can have concurrent immunosuppressive effects for dams (Smyth et al. 2016; Smyth et al. 2018) and delayed immunosuppressive effects for offspring may introduce a new level of complexity to the ICHH, namely, an intergenerational consequence of androgen‐mediated sexual selection operating in females.

## Author Contributions

**Kendra N. Smyth‐Kabay:** conceptualization (equal), data curation (lead), formal analysis (equal), funding acquisition (supporting), investigation (lead), methodology (lead), project administration (equal), supervision (equal), visualization (equal), writing – original draft (lead), writing – review and editing (supporting). **Nicholas M. Caruso:** formal analysis (equal), validation (lead), visualization (equal), writing – review and editing (supporting). **Alexandra C. Stonehill:** investigation (supporting), writing – review and editing (supporting). **Tim H. Clutton‐Brock:** funding acquisition (supporting), resources (lead), supervision (supporting), writing – review and editing (supporting). **Christine M. Drea:** conceptualization (equal), funding acquisition (lead), investigation (supporting), methodology (supporting), project administration (lead), resources (lead), supervision (lead), writing – original draft (supporting), writing – review and editing (lead).

## Conflicts of Interest

The authors declare no conflicts of interest.

## Statement on Inclusion

The field portion of our study was conducted at the Kalahari Meerkat Project (KMP), located at a remote site in the Kalahari Desert of South Africa. Fieldwork was discussed with and approved by the local stakeholders of the Kuruman River Reserve. As is the norm at the KMP, our fieldwork was conducted by a widely international and diverse team, including individuals based in South Africa, to whom we are indebted. The laboratory and analytical portion of our study was conducted in the United States; our publication brings together authors from different countries.

## Data Availability

Our data are available in the Open Science Framework repository, at the following link: https://osf.io/7gavc/?view_only=5c92636f08dd4425ba5601d009038a87.

## Appendix A

**TABLE A1:** Factors associated with parasitism (parasite species richness, PSR; faecal egg counts, FEC) and innate immune function (bacteria killing ability, BKA scores; haemolytic complement activity, HCA scores) in juvenile meerkat offspring, as determined by linear models (LMs) and linear mixed models (LMMs). Comparisons testing for effects of maternal category (dominant control (DC); subordinate control (SC); dominant treated (DT)) are made between the juvenile offspring of DC dams and those of SC or DT dams (under Predictor). Total sample sizes are indicated for each model (see Figure 2 for sample size breakdowns by maternal category).

Response (*n*)	Predictor	*F/χ* ^2^ ^a^ (df)	*p*	Estimate (SE)	Post hoc *p*
PSR^c^ (89)	SC	10.43 (2)	0.005	−0.74 (0.33)	0.058
	DT			−1.15 (0.33)	0.003
	Weight	20.69 (1)	< 0.001	−0.84 (0.24)	—
	SC: Weight	0.06 (2)	0.970	−0.06 (0.26)	—
	DT: Weight			−0.06 (0.39)	—
	Age	15.16 (1)	< 0.001	0.68 (0.18)	—
	Rain	1.20 (1)	0.157	0.15 (0.11)	—
FEC^c^ (71)	SC	10.08 (2)	0.007	−1.50 (0.67)	0.048
	DT			−1.80 (0.62)	0.004
	Weight	3.72 (1)	0.054	−0.60 (0.56)	—
	SC: Weight	1.90 (2)	0.388	−0.18 (0.73)	—
	DT: Weight			−1.03 (0.79)	—
	Age	5.08 (1)	0.024	0.91 (0.39)	
	Rain	0.003 (1)	0.960	0.03 (0.21)	—
BKA scores^b^ (44)	SC	5.87 (2, 38)	0.006	−10.62 (7.92)	0.320
	DT			34.43 (12.77)	0.020
	Weight	2.98 (1, 38)	0.092	4.70 (6.56)	—
	SC: Weight	1.04 (2, 38)	0.362	0.15 (8.03)	—
	DT: Weight			−23.08 (16.93)	—
HCA scores^b^ (28)	SC	0.001 (1, 24)	0.978	−13.39 (20.58)	—
	Weight	7.57 (1, 24)	0.011	−48.19 (15.34)	—
	SC: Weight	2.72 (1, 24)	0.112	38.77 (23.49)	—

^a^ *χ* ^2^ = likelihood ratio test statistic (LMMs).

^b^ Results of a LM.

^c^ Results of a LMM with individual as a random factor.

**TABLE A2:** Factors associated with parasitism (parasite species richness, PSR; faecal egg counts, FEC) and innate immune function (bacteria killing ability, BKA scores; haemolytic complement activity, HCA scores) in adult meerkat offspring as determined by linear mixed models (LMMs). Comparisons for maternal category (dominant control (DC); subordinate control (SC); dominant treated (DT)) are made between the adult offspring of DC dams and those of SC or DT dams (under Predictor). Sex comparisons are made against females. Total sample sizes are indicated for each model (see Table 2 for sample size breakdowns by maternal category).

Response (*n*)	Predictor	*χ* ^2^ ^a^ (df)	*p*	Estimate (SE)
PSR^b^ (109)	SC	2.28 (2)	0.320	−0.70 (0.39)
	DT			−0.26 (0.46)
	Sex	3.22 (1)	0.073	0.32 (0.27)
	Weight	1.32 (1)	0.251	−0.17 (0.15)
	Age	0.59 (1)	0.444	0.24 (0.26)
	Rain	0.32 (1)	0.569	−0.03 (0.11)
FEC^b^ (44)	SC	3.03 (2)	0.219	−1.38 (1.14)
	DT			−1.15 (1.22)
	Sex	2.44 (1)	0.118	0.77 (0.85)
	Weight	0.01 (1)	0.924	−0.22 (0.54)
	Age	0.002 (1)	0.962	0.27 (0.86)
	Rain	0.15 (1)	0.703	0.13 (0.28)
BKA score^b^ (67)	SC	0.32 (2)	0.853	0.01 (0.19)
	DT			−0.04 (0.20)
	Sex	4.71 (1)	0.030	−0.24 (0.16)
	Weight	0.31 (1)	0.577	−0.04 (0.08)
	Age	1.37 (1)	0.241	0.08 (0.08)
HCA score^b^ (65)	SC	1.86 (2)	0.394	−0.06 (0.10)
	DT			−0.01 (0.08)
	Sex	2.52 (1)	0.113	−0.03 (0.08)
	Weight	0.21 (1)	0.648	0.01 (0.04)
	Age	0.001 (1)	0.994	−0.02 (0.04)

^a^ *χ* ^2^ = likelihood ratio test statistic (LMMs).

^b^ Results of a LMM with individual identity nested within group identity as a random factor.

**TABLE A3:** Relationships between innate immune function (bacteria killing ability, BKA scores; haemolytic complement activity, HCA scores) and sex hormone concentrations (androstenedione, A_4_; testosterone, T; oestradiol, E_2_) in the juvenile meerkat offspring from dominant control and subordinate control mothers, as determined by linear models.

Immune measure	Hormone	Estimate (SE)	*F* (df)	*p*
BKA score	A_4_	−0.03 (0.08)	0.11 (1, 38)	0.738
	T	−0.03 (0.08)	0.12 (1, 35)	0.735
	E_2_	0.09 (0.08)	1.28 (1, 32)	0.267
HCA score	A_4_	−6.18 (10.28)	0.36 (1, 25)	0.553
	T	−15.36 (9.48)	2.63 (1, 24)	0.118
	E_2_	6.73 (11.85)	0.32 (1, 21)	0.576
